# Chronic Oral Palmitoylethanolamide Administration Rescues Cognitive Deficit and Reduces Neuroinflammation, Oxidative Stress, and Glutamate Levels in A Transgenic Murine Model of Alzheimer’s Disease

**DOI:** 10.3390/jcm9020428

**Published:** 2020-02-05

**Authors:** Sarah Beggiato, Maria Cristina Tomasini, Tommaso Cassano, Luca Ferraro

**Affiliations:** 1 Department of Life Sciences and Biotechnology, University of Ferrara, 44121 Ferrara, Italy; bggsrh@unife.it (S.B.); tmsmcr@unife.it (M.C.T.); 2 Department of Medical, Oral and Biotechnological Sciences, University of Chieti-Pescara, 66100 Chieti, Italy; 3 IRET Foundation, Ozzano Emilia, 40064 Bologna, Italy; 4 Department of Clinical and Experimental Medicine, University of Foggia, 71122 Foggia, Italy; tommaso.cassano@unifg.it; 5 Technopole of Ferrara, LTTA Laboratory for the Technologies for Advanced Therapies, 44121 Ferrara, Italy

**Keywords:** Alzheimer’s disease, cognitive dysfunctions, extracellular glutamate levels, reactive oxygen species, synaptophysin, hippocampus

## Abstract

N-palmitoylethanolamide (PEA) is a lipid mediator belonging to the class of the N-acylethanolamine. Products containing PEA, also in ultramicronized formulation (um-PEA), are already licensed for use in humans for its analgesic and anti-inflammatory properties, and demonstrated high safety and tolerability. Preclinical studies indicate that PEA, especially in the ultramicronized form, could be a potential therapeutic agent for Alzheimer’s disease (AD). In this study, we evaluated the neuroprotective and antioxidant effects of chronic (three months) um-PEA administration in an animal model of AD (3×Tg-AD mice). For translation purposes, the compound has been orally administered. Cognitive performance as well as biochemical markers [(interleukin-16 (IL-16) and tumor necrosis factor-α (TNF-α)] levels, reactive oxygen species (ROS) production, synaptophysin and glutamate levels) have been evaluated at the end of um-PEA treatment. The results indicate that orally administered um-PEA was adsorbed and distributed in the mice brain. The chronic treatment with um-PEA (100 mg/kg/day for three months) rescued cognitive deficit, restrained neuroinflammation and oxidative stress, and reduced the increase in hippocampal glutamate levels observed in 3×Tg-AD mice. Overall, these data reinforce the concept that um-PEA exerts beneficial effects in 3×Tg-AD mice. The fact that PEA is already licensed for the use in humans strongly supports its rapid translation in clinical practice.

## 1. Introduction

Alzheimer’s disease (AD) is the most prevalent neurodegenerative disorder in the world, accounting for more than 80% of dementia cases in elderly people. The pathology leads to the progressive loss of mental and learning abilities, functional decline, as well as to behavioral disorders and neuropsychiatric symptoms [1,2,3]. Extracellular neuritic β-amyloid peptide (Aβ), creating senile plaques, and intracellular neurofibrillary tangles due to tau hyperphosphorylation occupating much of the cytoplasm of pyramidal neurons as well as of other cells, are considered as hallmarks of the AD brain [4]. More recent findings clearly support the view that neuroinflammation and oxidative stress play a critical role in the pathophysiology of AD [5,6,7,8]. Increased glial cell activation and oxidative stress are reliably observed in rodent models of AD as well [9,10]. As a consequence, a therapeutic approach targeting not only neurodegeneration, but also neuroinflammation and oxidative stress may prove effective in slowing the progression of the disease [11,12,13]. In this context, palmitoylethanolamide (PEA), an endogenous lipid mediator [14], seems to be a promising pharmacological agent. In fact, the compound has been demonstrated effective in reducing neuroinflammation and neurodegeneration in several in vitro and in vivo models of AD [15]. We previously demonstrated that PEA (0.1 μM) exerts a neuroprotective effect against Aβ_1-42_ fragment (Aβ_42_)-induced toxicity in primary cultures of cortical neurons or astrocytes from wild-type (non-Tg) and from a triple-transgenic murine model of AD (3×Tg-AD mice) [16]. More recently, by using a mouse astrocyte-neuron co-culture preparation, we also proved that astrocytes contribute to Aβ_42_-induced neurotoxicity and PEA (0.1 μM), by blunting Aβ_42_-induced astrocyte activation, improves neuronal survival [17]. These findings indicate that PEA exerts beneficial effects against Aβ-induced neurodegeneration, not only by a direct action at the neuronal level, but also by reducing the deleterious consequences of astrocyte dysfunctions. Most of these effects are due to the ability of PEA to act as a peroxisome proliferator-activated receptor (PPAR)-α agonist with an EC_50_ value of 3.1 ± 0.4 μM [18,19]. In line with these in vitro findings, in vivo evidence demonstrated that chronic administration of PEA in ultramicronized formulation (um-PEA; i.e., a formulation associated with high PEA bioavailability) [14,20,21] reduced Aβ formation and phosphorylation of tau protein and promoted neuronal survival in the CA1 subregion of the hippocampus. These effects were associated with a normalization of the astrocytic function and of reduced hippocampal levels of glutamate-glutamine complex (Glx; a combined measure of glutamate and glutamine), as well as with a general reduction of neuroinflammatory conditions [22]. Although accumulating findings support the anti(neuro)inflammatory properties of PEA, at the present there are no data on the possible involvement of antioxidant mechanisms in the beneficial actions of PEA against neurodegenerative processes.

In this study, we evaluated the possible neuroprotective and antioxidant effects of chronic (3 months) um-PEA administration in 3×Tg-AD mice at a mild stage of AD-like pathology and cognitive deficits (five months of age). For translation purposes, we chose to orally administer the compound by including it in the animal’s food. The possible involvement of anti-(neuro)inflammatory and antioxidant mechanisms in the PEA-induced effects as well as cognitive performances were evaluated at the end of um-PEA treatment. For treatment, 3×Tg-AD mice were chosen because this line is probably the genetic mouse model with the highest face and construct validity [23]. These animals represent a widely used and validated model which closely mimics the neuropathological alterations seen in human AD [24,25], although results in AD mouse models to date have not been shown to have high predictive validity [26]. In particular, 3×Tg-AD mice develop age-related, progressive neuropathology including plaques and tangles. Extracellular Aβ deposits are apparent by six months in the frontal cortex, and become more extensive by twelve months. Although tau pathology is not observed at six months, it is evident by twelve months. Synaptic dysfunction, including LTP deficits, occurs prior to plaques and tangles. Furthermore, cognitive deficits are evident at four months of age [27], while oxidative stress precedes these impairments, comprising some of the earliest pathological signs noted in this animal model of AD [28,29].

Overall, the results reinforce the concept that um-PEA exerts anti(neuro)inflammatory and antioxidant activities, thus inducing therapeutic effects in 3×Tg-AD mice and ameliorating both cognitive deficits and a range of neuropathological features.

## 2. Materials and Methods

### 2.1. Animals

Colonies of 3×Tg-AD mice (harboring APPswe, PS1M146V, and tauP301L transgenes) and wild type littermates (non-Tg mice; i.e., controls) were used. Male 3×Tg-AD mice and their sex- and age-matched wild-type littermates (C57BL6/129SvJ) were maintained in controlled conditions (12-h light/12-h dark cycle, temperature 22 °C, humidity 50–60%, fresh food, and water ad libitum) at the animal facilities of the Puglia and Basilicata Experimental Zooprophylactic Institute (Foggia, Italy) [30]. Genotypes were confirmed by polymerase chain reaction (PCR) after tail biopsies, according to the procedures described previously [24,25].

The experimental protocols performed in this study were in accordance with the European Communities Council Directive of September 2010 (2010/63/EU) and were approved by the Italian Ministry of Health. Efforts were made to minimize the number of animals used and to reduce their discomfort.

### 2.2. Pharmacokinetic Studies

#### 2.2.1. Single Oral Administration of Um-PEA

Oral um-PEA (please refer to [20] for details of compound preparation) blood absorption and brain distribution have been evaluated before to perform the chronic treatment study. To this purpose, the time-dependence of plasma and brain tissue levels of PEA in healthy (i.e., non-Tg) mice were measured by orally administering a single dose (100 mg/kg body weight) of commercial um-PEA dissolved in vehicle [water/PEG/Tween-80 (90/5/5 ratio), *v/v*)]; [18]. Parallel groups of mice were administered with the vehicle. The compound or the vehicle was given by oral gavage, using flexible tubes to reduce animal stress. Mice were sacrificed by anesthetic (isoflurane) overdose before or 1, 1.5, 3 or 4 h after um-PEA or vehicle administration (*n* = 5/time point). Blood as well as hippocampus and prefrontal cortex (PFC) collected at sacrifice were immediately frozen in liquid nitrogen and stored at −80 °C for later PEA analysis. Plasma and tissue PEA levels were measured as described by Sharma et al. [31] and Liput et al. [32], respectively.

#### 2.2.2. Sub-Chronic Oral Administration of Um-PEA

The effects of um-PEA (100 mg/kg body weight) oral (gavage) administration on plasma and brain tissue levels of PEA were also measured in non-Tg mice previously fed with the compound (100 mg/kg/day) for 8 consecutive days. We first determined that each mouse ate approximately 4 g/day of standard rodent chow (Mucedola S.R.L., Italy). Rodent chow was ground finely in a food processor and one week prior the initiation of the treatment, mice were acclimated to a wet mash diet. Beginning of the treatment, um-PEA (100 mg/kg body weight) was thoroughly mixed into the food daily for PEA-treated mice, while controls continued to receive wet mash alone. The treatment duration was 8 days, the animals were single-housed and on the last day the compound or the vehicle was given by oral gavage. Blood, hippocampus, and PFC PEA levels at different time-points were determined as described above.

### 2.3. Effects of A Chronic (3 Months) Treatment with Um-PEA on Cognitive Performance and Biochemical Parameters

#### 2.3.1. Animal Treatment

To evaluate the possible neuroprotective and/or antioxidant properties of um-PEA, age-matched non-Tg mice and 3×Tg-AD mice (2 months ± 2 weeks of age) have been orally treated for 3 months with the compound (100 mg/kg/day). To avoid the possible induction of stress to the animals as a consequence of daily *gavage* for 3 months, in the chronic study um-PEA had been administered through animal food, as described above. Both non-Tg and 3×Tg-AD mice were randomly assigned to either standard (i.e., controls) or PEA-enriched diet. No animals were excluded from the analysis. Mice were regularly weighed during the entire period of the treatment.

Behavioural and biochemical studies were conducted at the end of the 3-month treatment (animal age = 5 months ± 2 weeks).

#### 2.3.2. Behavioral Test: Novel Object Recognition Test

Mouse cognitive performance was assessed utilizing the novel object recognition (NOR) test at the end of the treatment period. The experiments were performed between 8:00 a.m. and 3:00 p.m., in a dimly lit condition and as previously described [22]. Briefly, after a 60 min of acclimation period in the behavioral room [an empty Plexiglas arena (45 ×  25  × 20 cm) for 3 consecutive days], mice were exposed to two identical objects (A + A) placed at opposite ends of the arena for 5 min. The mice were then subjected to a 5-min retention session after 30 min and 24 h. During these sessions, the mice were exposed to one object A and to a novel object B (30 min) or object C (24 h). Exploration was considered as pointing the head toward an object at a distance of <2.5 cm from the object, with its neck extended and vibrissae moving. Turning around, chewing, and sitting on the objects were not considered exploratory behaviors. Behavior was recorded with a MV750i camera (1024  × 768 resolution, Canon, Tokyo, Japan) and scored by a blinded investigator. Videotapes were analyzed as MPEG files using a behavioral tracking system furnished with infrared lighting-sensitive CCD cameras. Animal performances were monitored with the EthoVision XT version 7 video-tracking software system (Noldus Information Technology Inc., Leesburg, VA, USA). The time of exploration was recorded, and an object recognition index (ORI) was calculated, such that ORI = (TN − TF)/(TN  +  TF), where TN and TF represent times of exploring the familiar and novel object, respectively. Mice that did not explore both objects during training were discarded from further analysis.

#### 2.3.3. Biochemical Analyses

##### TNF-α and IL-16 levels

Anti(neuro)inflammatory effects of chronic um-PEA treatment have been evaluated by measuring the levels of two pro-inflammatory cytokines such as tumor necrosis factor alpha (TNF-α) and interleukin 16 (IL-16). In particular, we measured: 1) plasma TNF-α levels by using a mouse TNF-α KIT (AlphaLISA, AL505C, PerkimmElmer^®^ Italy, Milan, Italy); 2) TNF-α and IL-16 levels in hippocampal homogenates by using a mouse cytokine array panel A (R&D Systems, Minneapolis). A total of 100 μg for each hippocampal lysate were processed following the manufacturer’s instructions [22].

##### Measurement of Reactive Oxygen Species

Reactive oxygen species (ROS) were determined in the hippocampus of um-PEA treated and untreated mice to determine whether there was any evidence for compound-induced antioxidant effects. To this purpose, hippocampal homogenates (100 μg/100 μL) were added to the wells of a 96-well plate and treated with 30 μM of 2′,7′-dichlorofluorescin diacetate (DCFH-DA). Changes in fluorescence intensity (due to the conversion from DCFH-DA to dichlorofluorescein) were measured every 5 min for 30 min after DCFDA treatment using a Multimode Microplate Reader (EnSight™ Multimode Microplate Reader, PerkinElmer) at excitation and emission wavelengths of 485 nm and 530 nm, respectively [33].

##### Synaptophysin Levels

The synaptic marker synaptophysin was determined in the hippocampus of um-PEA treated and untreated mice to determine whether there was any evidence for compound-induced neuroprotection. Animals were sacrificed and hippocampi rapidly dissected out, frozen on dry ice, and stored at −80 °C until analysis. Tissues were homogenized in lysis buffer (RIPA buffer, protease inhibitor cocktail) and centrifuged at 12,000 rpm at 4 °C for 30 min. Supernatants were collected and protein levels were quantified by western blot analysis using the bicinchoninic acid protein assay kit (Thermo Fisher Scientific, Inc., Waltham, MA). Forty micrograms of protein per sample were separated on a 4% to 12% gradient polyacrilamide precast gels (Novex WedgeWell 4–12% Tris-Glycine Gels; Life Technologies, Carlsbad, CA, USA) in a Bolt Mini Gel Tank apparatus (Life Technologies). Proteins were then transferred onto polyvinyl difluoride membrane, blocked for 60 min with 5% nonfat dry milk in 0.1% Tween 20 Tris-buffered saline, and incubated overnight at 4 °C with rabbit anti-synaptophysin polyclonal antibody (catalog no.: # 4329, 1: 1000; Cell Signaling Technology, Danvers, MA, USA). Mouse anti-β-tubulin monoclonal antibody (catalog no.: # BT7R, 1:3000; Life Technologies) was used as loading control. Membranes were washed, then incubated for 1 h at room temperature with horseradish peroxidase-linked secondary antibodies (catalog no.: # 12–348, 1:5000; Merck Millipore). Membrane were incubated with a specific secondary horseradish peroxidase (HRP)-conjugated antibody goat anti-rabbit IgG (catalog no., #GTXRB-003, 1:2000, Microtech Research Products, Raleigh, NC, USA); HRP-conjugated goat anti-mouse IgG, (catalog no., # A16072, 1:5000, Life Technologies), either in nonfat dry milk or BSA TBS-T. Immunocomplexes were detected by enhanced chemiluminescence (ECL) kit.

Immunoreactivity was visualized by an enhanced chemiluminescence detection kit (catalog no., # 34079, SuperSignal West Pico Trial Kit, Life Sciences) and the signal obtained was quantified by ImageJ Software after densitometric scanning of the X-ray films (catalog no., # RX1824 * A, Fuji Film, Milan, Italy). The optical density of specific synaptophysin bands was normalized to the corresponding tubulin levels.

##### Extracellular Glutamate Levels in Mouse Hippocampus

In vivo microdialysis was used to analyse extracellular glutamate levels in the hippocampus of mice fed with standard or PEA-enriched diet. A CMA/7 guide cannula with stylet (CMA Microdialysis, Stockholm, Sweden) was stereotaxically implanted into the ventral hippocampus (stereotaxic coordinates: A = −3.0 mm; L = ± 3.0 mm; V = −1.8 mm from bregma) of anesthetized mice. 36 h later, a CMA/7 probe was inserted, connected to a microperfusion pump (CMA 100; Carnegie Medicin, Stockholm, Sweden) set to a speed of 1 μL/min and perfused with an artificial cerebrospinal fluid consisting of (in mM): NaCl (122), KCl (3), CaCl_2_ (1.3), MgSO_4_ (1.2), NaHCO_3_ (25), and KH_2_PO_4_ (0.4) [34]. After a 2-hour stabilization period, four baseline samples were collected every 20 min. Probe position was verified histologically and glutamate was quantified by HPLC coupled to fluorescence detection as previously described [34].

### 2.4. Statistical Analysis

All data are expressed as mean ± standard error of measurement (SEM). Behavioral and biochemical data were analyzed by two-way analyses of variance (ANOVA) with genotype (3×Tg-AD vs. non-Tg) and treatment (um-PEA vs. placebo) as between-subject factors. Tukey′s honestly significant difference (HSD) test or Bonferroni’s test were used for multiple post hoc comparisons when required. Student *t*-test was also used when appropriate. The threshold for statistical significance was set at *p*  <  0.05.

## 3. Results

### 3.1. Basal Levels of PEA in Plasma and Brain Homogenates of Non-Tg and 3×Tg-AD Mice

Mean basal plasma levels of PEA in non-Tg mice were 31.45 ± 5.39 pmol/mL and were similar to those measured in 3×Tg-AD mice (28.75 ± 6.16 pmol/mL). In the hippocampal tissue from 3×Tg-AD mice a tendency to a decrease in mean basal PEA levels was observed in respect to non-Tg mice levels (134 ± 26 pmol/g and 176 ± 24 pmol/g, respectively; *p* = 0.062; Student *t*-test). Finally, PFC mean basal PEA levels were similar in non-Tg mice (116 ± 21 pmol/g) and 3×Tg-AD mice (108 ± 17 pmol/g).

### 3.2. Levels of PEA in Plasma and Brain Homogenates of Non-Tg Mice after A Single Oral Administration of Um-PEA

**Figure 1:**
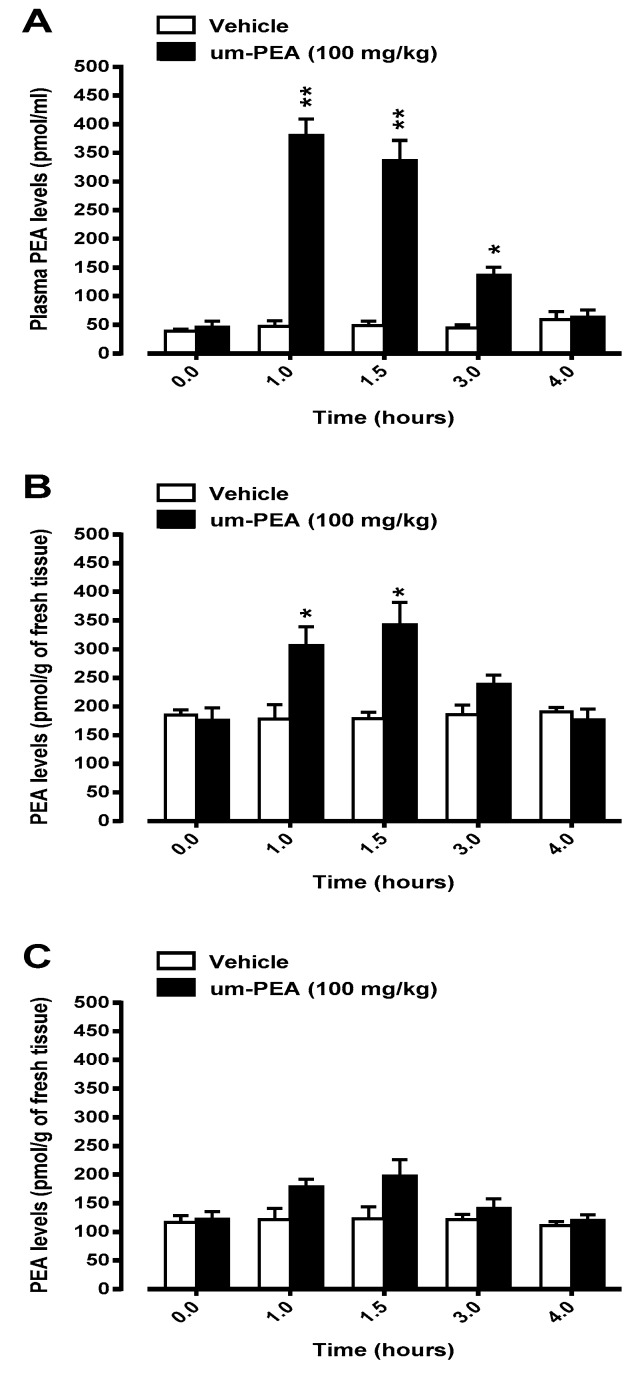
Levels of palmitoylethanolamide (PEA) in plasma (*panel **A***), hippocampus (*panel **B***) and prefrontal cortex (*panel **C***) of non-Tg mice 1, 1.5, 3, and 4 hours after oral administration (by *gavage*) of 100 mg/kg ultramicronized palmitoylethanolamide (um-PEA) or its vehicle [water/PEG/Tween-80 (90/5/5 ratio), *v/v*)]. Data are means ± SEM of five animals for each group. * *p* < 0.05; ** *p* < 0.01 significantly different from the respective vehicle time-point (Student *t*-test).

The concentrations of PEA in plasma as well as in hippocampus and PFC homogenates were measured at 0, 1, 1.5, 3, and 4 h after a single oral administration of um-PEA (100 mg/kg body weight). In non-Tg mice treated with um-PEA, mean basal plasma levels of PEA were significantly higher than those measured in vehicle treated animals. In particular, a significant peak concentration of PEA was found 1 h after the compound administration, being ~9 times higher than basal values. PEA plasma levels dropped 4 h after um-PEA administration to concentrations comparable to the basal ones (Figure 1A). No significant changes in mean basal plasma levels of PEA were measured in vehicle-treated non-Tg mice (Figure 1A).

We next assessed hippocampus and PFC levels of PEA after a single oral dose of um-PEA (100 mg/kg body weight). In the hippocampal tissue from non-Tg mice, significantly higher PEA levels were observed 1 and 1.5 h after a single oral administration of um-PEA (Figure 1B), while a trend to increased PEA levels was observed at the 3 h time-point. PEA hippocampal levels dropped 4 h after um-PEA administration to concentrations similar to those measured in vehicle-treated mice (Figure 1B). The oral administration of um-PEA (100 mg/kg body weight) was associated with a trend to a rise in PFC PEA levels at 1 and 1.5 h time-points, but these increases did not reach the statistical significance (Figure 1C). No significant changes in mean basal hippocampus or PFC levels of PEA were measured in vehicle-treated non-Tg mice (Figure 1B,C, respectively).

### 3.3. Levels of PEA in Plasma and Brain Homogenates of Non-Tg Mice after A Sub-Chronic Oral Administration of Um-PEA

The effects of um-PEA (100 mg/kg body weight) oral administration on plasma and brain tissue levels of PEA were measured in non-Tg mice previously fed with the compound (100 mg/kg/day) for 8 consecutive days. Under these experimental conditions, the administration of um-PEA (100 mg/kg body weight) induced rises in mean plasma, hippocampus and PFC PEA levels, which were similar to those observed after the single oral administration of um-PEA in naïve non-Tg mice.

### 3.4. Effects of A Chronic (3 Months) Treatment with Um-PEA on Cognitive Performance and Biochemical Parameters in Non-Tg and 3×Tg-AD Mice

The possible beneficial effects of a chronic um-PEA treatment have been evaluated in pre-symptomatic 3×Tg-AD mice by evaluating some cognitive and neuropathological parameters. To this aim, age-matched non-Tg mice and 3×Tg-AD mice (2 months ± 2 weeks of age) have been randomly assigned to treatment (um-PEA 100 mg/kg/day) or control groups. Mice group-housed received a special diet supplemented with um-PEA or a control standard diet for 3 months. At the end of the treatment, the animals underwent cognitive tests or were sacrificed to evaluate biochemical parameters in the hippocampus.

Mice have been weighed prior the beginning of dietary regimen and regularly every three weeks. No body weight gain differences were observed between groups and all the animals completely ate the daily presented food.

#### 3.4.1. Um-PEA Improves learning and Memory in 5-Month-old 3×Tg-AD Mice

**Figure 2:**
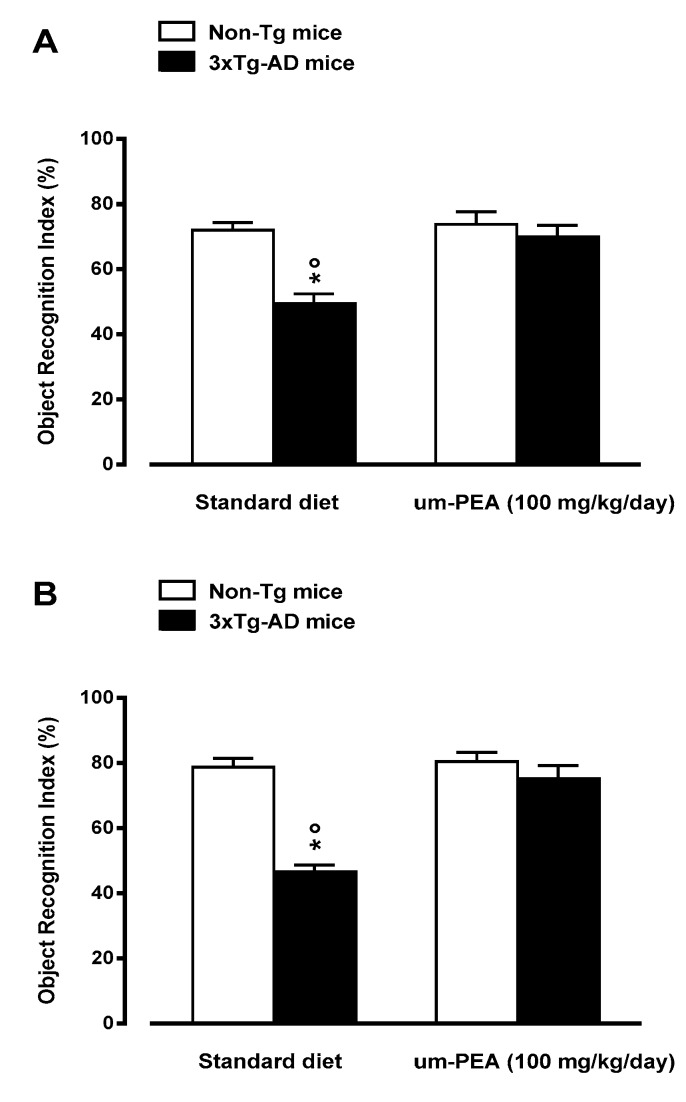
Oral ultramicronized palmitoylethanolamide (um-PEA) treatment rescues short-(3 h; *panel **A***) and long-term (24 h; *panel **B***) memory deficits in the 3×Tg-AD mice, as evaluated by novel object recognition test. Age-matched non-Tg mice and 3×Tg-AD mice (2 months ± 2 weeks of age) have been orally treated for 3 months with the compound (100 mg/kg/day, added to the animal food), while control animals were fed with a standard diet during the treatment period. Novel object recognition test was performed at the end of the treatment period. The data are presented as means ± SEM of 9–11 animals for each group. * *p* < 0.05 significantly different from the respective non-Tg mouse group; *p* < 0.05 significantly different from the respective PEA-treated group according to two-way ANOVA followed by Tukey multiple-comparison test.

At the end of the three month treatment, the effects of um-PEA on both short- (30 min) and long-term (24 h) memory were evaluated by using the NOR test. Two-way ANOVA analysis revealed significant changes in the time mice spent exploring the new object across the four different groups. At 30 min, we found significant genotype-by-treatment interaction effects, while no significant differences were found for the main effects of genotype and treatment. Post-hoc comparisons showed a significant higher object recognition index (ORI) for 3×Tg-AD mice fed with um-PEA with respect to standard diet-fed 3×Tg-AD mice (Figure 2A). Performing this trial 24 h later, we observed a significant genotype and genotype-by-treatment interaction effect of ORI in the exploration session among the four groups. Post-hoc analysis demonstrated that um-PEA-fed 3×Tg-AD mice performed significantly better than the standard diet-fed 3×Tg-AD group (Figure 2B), while, at both time points, um-PEA had no effect on the performance of non-Tg mice (Figure 2).

#### 3.4.2. Um-PEA Partially Restrains Neuroinflammation

**Figure 3:**
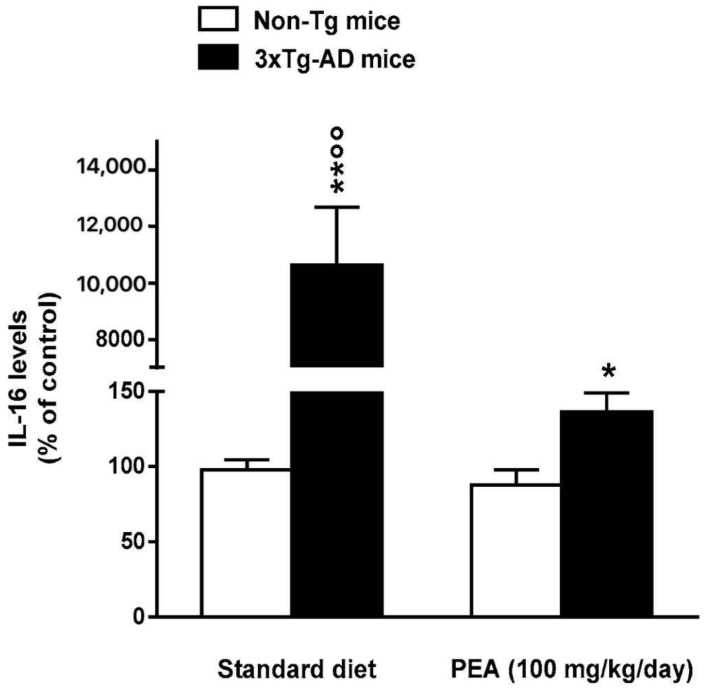
Effects of oral ultramicronized palmitoylethanolamide (um-PEA) treatment on interleukin 16 (IL-16) levels in the hippocampus of non-Tg and 3×Tg-AD mice. Age-matched non-Tg mice and 3×Tg-AD mice (2 months ± 2 weeks of age) have been orally treated for 3 months with the compound (100 mg/kg/day, added to the animal food), while control animals were fed with a standard diet during the treatment period. Biochemical analyses were performed on hippocampus tissues taken from mouse brains at the end of the treatment period. The data are expressed as percentage ± SEM of control (standard diet fed non-Tg mice) (*n* = 4, in triplicate). * *p* < 0.05, ** *p* < 0.01 significantly different from the respective non-Tg mouse group; *p* < 0.01 significantly different from the respective PEA-treated group according to two-way ANOVA followed by Tukey multiple-comparison test.

**Figure 4:**
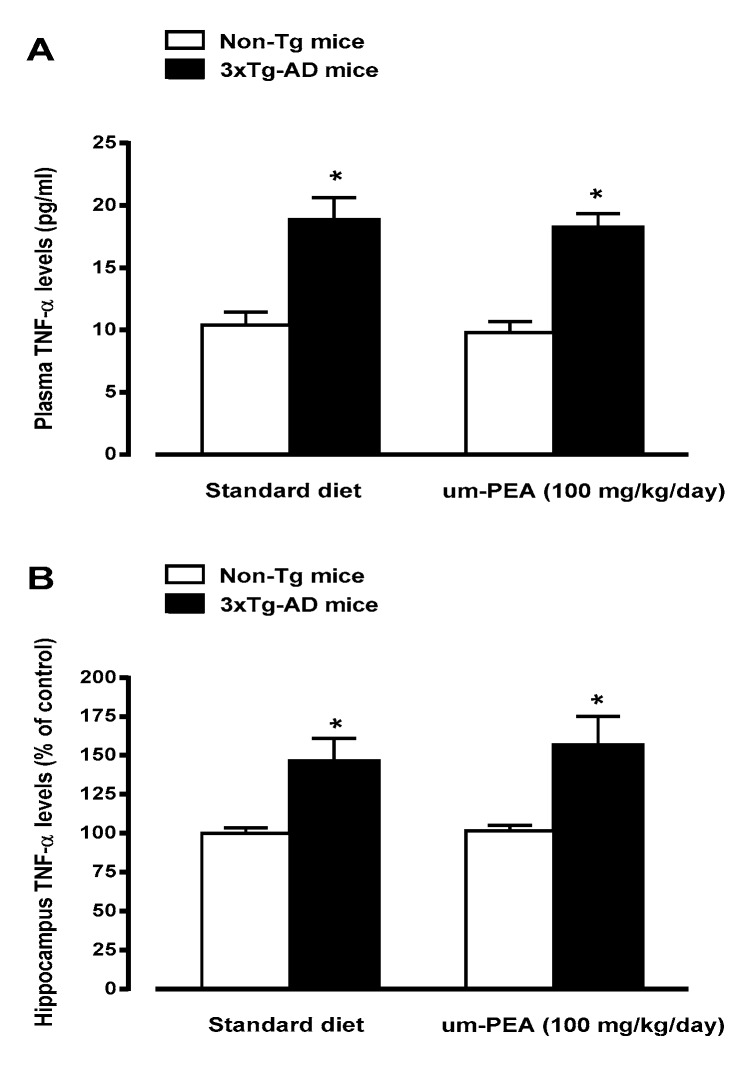
Effects of oral ultramicronized palmitoylethanolamide (um-PEA) treatment on plasma (*panel **A***) and hippocampus (*panel **B***) tumor necrosis factor alpha (TNF-α) levels in non-Tg and 3×Tg-AD mice. Age-matched non-Tg mice and 3×Tg-AD mice (2 months ± 2 weeks of age) have been orally treated for 3 months with the compound (100 mg/kg/day, added to the animal food), while control animals were fed with a standard diet during the treatment period. Biochemical analyses were performed on hippocampus tissues taken from mouse brains at the end of the treatment period. The data are presented as absolute values (*panel **A***) or as percentage ± SEM of control (standard diet-fed non-Tg mice; *Panel **B***) (*n* = 4, in triplicate). * *p* < 0.05 significantly different from the respective non-Tg mouse group according to two-way ANOVA followed by Tukey multiple- comparison test.

The levels of TNF-α and IL-16, two pro-inflammatory cytokines, were measured in plasma and/or in the hippocampus of all groups. Standard diet-fed 5-month-old 3×Tg-AD mice displayed TNF-α and IL-16 overproduction in respect to non-Tg mice, thus suggesting that 3×Tg-AD mice exhibit a transition to an increased pro-inflammatory state. Chronic treatment with um-PEA almost completely abolished the increase in hippocampus IL-16 (Figure 3), but had no effects on the enhancements in plasma and hippocampus TNF-α expression (Figure 4A,B). The treatment with um-PEA had no effect on TNF-α and IL-16 levels in non-Tg mice (Figure 3; Figure 4).

#### 3.4.3. Um-PEA Reduces ROS Production

**Figure 5:**
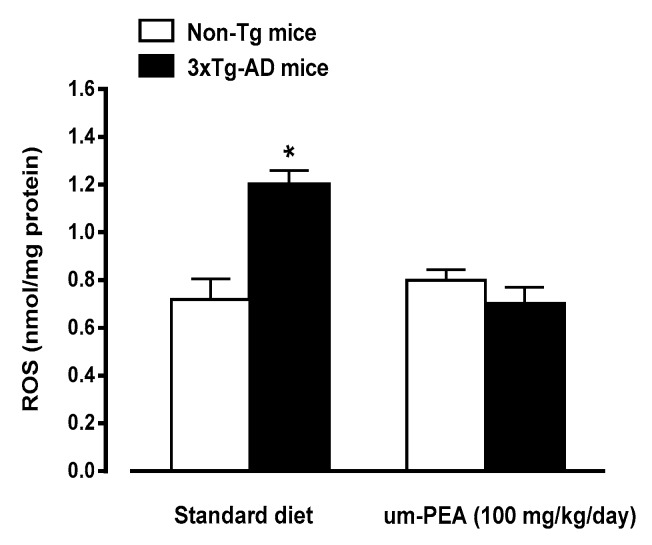
Effects of oral ultramicronized palmitoylethanolamide (um-PEA) treatment on reactive oxygen species (ROS) production in hippocampus homogenates from non-Tg and 3×Tg-AD mice. Age-matched non-Tg mice and 3×Tg-AD mice (2 months ± 2 weeks of age) have been orally treated for 3 months with the compound (100 mg/kg/day, added to the animal food), while control animals were fed with a standard diet during the treatment period. ROS production was measured in hippocampus tissues taken from mouse brains at the end of the treatment period. The data are presented as mean ± SEM (*n* = 5, in triplicate). * *p* < 0.05 significantly different from the other groups according to two-way ANOVA followed by Tukey multiple-comparison test.

Considering the relevance of oxidative stress in AD, the production of ROS was evaluated in hippocampal homogenates obtained from all groups under investigation. Detection of basal ROS production was modestly, but significantly, higher in standard diet-fed 3×Tg-AD mice than in both um-PEA-treated or -untreated non-Tg mice (Figure 5). Interestingly, um-PEA significantly reduced basal ROS production in 3×Tg-AD mice, down to the control (i.e., non-Tg mice) levels.

#### 3.4.4. Um-PEA did not Affect Synaptophysin Levels in the Hippocampus of 3×Tg-AD Mice

**Figure 6:**
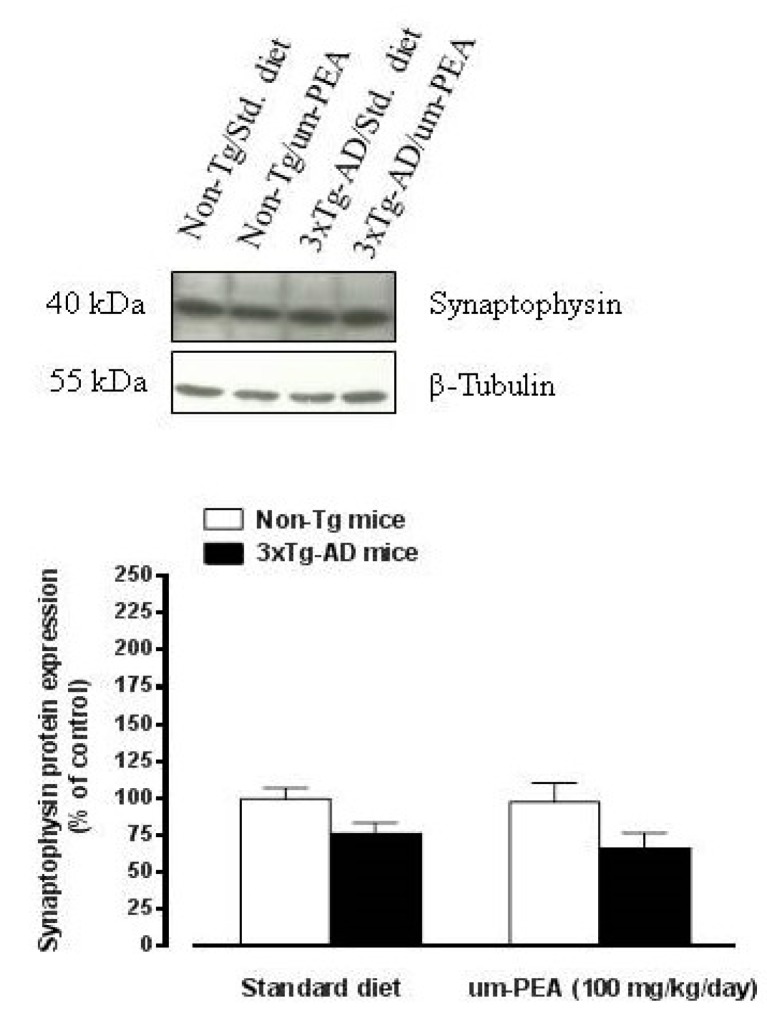
Oral ultramicronized palmitoylethanolamide treatment did not affect synaptophysin levels in the hippocampus of non-Tg and 3×Tg-AD mice. Age-matched non-Tg mice and 3×Tg-AD mice (2 months ± 2 weeks of age) have been orally treated for 3 months with the compound (100 mg/kg/day, added to the animal food), while control animals were fed with a standard diet during the treatment period. Representative western blot densitometric analysis of synaptophysin is reported above the graph. Western blot analysis was performed on hippocampus tissues taken from mouse brains at the end of the treatment period. Experiments were performed three times in triplicate and β-Tubulin was used as loading control. Results are expressed as percentage of the mean ± SEM of control value (i.e., standard diet fed non-Tg mice).

To evaluate whether chronic um-PEA treatment was associated with neuroprotection, the synaptic marker synaptophysin was determined in the hippocampus of all groups under investigation. A slight, not significant, decrease in hippocampal levels of synaptophysin was observed in 3×Tg-AD mice compared with non-Tg controls (Figure 6). The treatment with um-PEA did not affect the trend to a reduction of synaptophysin levels observed in 3×Tg-AD mice, and had no effect on the synaptic marker levels in the hippocampus of non-Tg mice (Figure 6).

#### 3.4.5. Um-PEA Rescues Increased Glutamate Levels in the Hippocampus of 3×Tg-AD Mice

**Figure 7:**
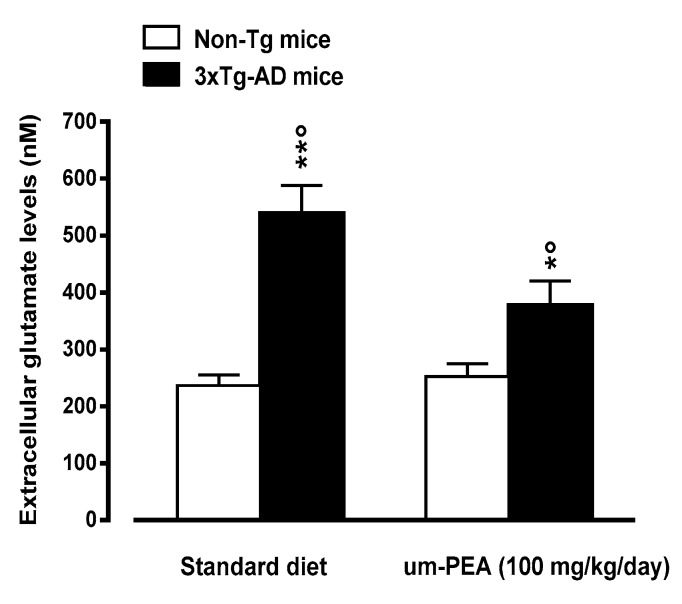
Oral ultramicronized palmitoylethanolamide (um-PEA) treatment rescues increased glutamate levels in the hippocampus of 3×Tg-AD mice. Age-matched non-Tg mice and 3×Tg-AD mice (2 months ± 2 weeks of age) have been orally treated for 3 months with the compound (100 mg/kg/day, added to the animal food), while control animals were fed with a standard diet during the treatment period. Microdialysis experiments were performed at the end of the treatment period. The data are presented as mean ± SEM of 6–7 animals/group. * *p* < 0.05, ** *p* < 0.01 significantly different from the respective non-Tg mouse group; *p* < 0.05 significantly different from the respective PEA-treated group according to two-way ANOVA followed by Tukey multiple-comparison test.

Microdialysis studies demonstrated that standard diet-fed five-month-old 3×Tg-AD mice had increased basal extracellular glutamate levels in the hippocampus, when compared to age-matched non-Tg mice (Figure 7). The treatment with um-PEA induced a significant reduction in hippocampal extracellular glutamate levels of 3×Tg-AD mice, although failing to restore control (i.e., non-Tg mice) levels. The treatment with um-PEA had no effects on extracellular glutamate levels in the hippocampus of non-Tg mice (Figure 7).

## 4. Discussion

This multidisciplinary study provides the first evidence that a chronic oral um-PEA treatment exerts anti-inflammatory and antioxidant effects in a murine model of AD that recapitulates most of the salient neural and cognitive impairments seen in this pathology [23,35]. The present results confirm recent in vivo findings suggesting that PEA could exert therapeutic properties in AD either by inducing neuroprotection or by reducing the neuroinflammation [15] and demonstrate, for the first time, the relevance of antioxidant effects for the ability of the compound to contrast AD-like symptoms in an animal model of the pathology. Finally, this study showed that 3×Tg-AD mouse plasma and brain PEA levels were slightly lower than those measured in non-transgenic animals. Although these differences were not significant, these findings lead to the suggestive hypothesis of the involvement of lower PEA levels in the etiopathogenesis of AD.

It has been recently reported that um-PEA, a crystalline form on micrometric size of PEA, is associated with enhanced solubility and potentially higher bioavailability than traditional formulations [14,21]. In particular, oral administration of um-[^13^C]_4_-PEA to healthy rats resulted in [^13^C]_4_-PEA detectable in the bloodstream already after 5 min, with a peak plasma concentration of 5.4 ± 1.87 pmol/mL. On the contrary, significant peak plasma concentration of [^13^C]_4_-PEA was not observed following the administration of naïve [^13^C]_4_-PEA [21]. The higher absorption of um-PEA than the naïve formulation of the compound has been also demonstrated in an animal model of acute inflammation (carrageenan-induced rat paw edema) [21] and was associated with a superior oral efficacy of um-PEA compared to naïve PEA [20]. Based on these findings, in a previous study we tested the anti-inflammatory and neuroprotective effects of a chronic treatment with um-PEA administered via a subcutaneous delivery system in 3×Tg-AD mice [22]. Overall, the results demonstrated that um-PEA improves learning and memory, reduces Aβ formation as well as phosphorylation of tau proteins, rescues impaired glutamatergic transmission, restrains neuroinflammation, and promotes neuronal survival in the CA1 subregion of the hippocampus of 3×Tg-AD mice. As oral delivery of drugs remains the most common route of administration, given its versatility, simplicity of administration and patient compliance, for translation purposes we decided to evaluate whether um-PEA maintains its possible therapeutic properties when orally administered. Moreover, to provide further insights into the role of the compound in counteracting AD pathology, the possible antioxidant effects of um-PEA in 3×Tg-AD mice were also investigated.

As there were no pharmacokinetic data on um-PEA after its oral administration to mice, we initially measured the absorption of um-PEA and its ability to reach central tissues after an acute oral administration of the compound. We observed that the oral um-PEA administration (100 mg/kg) was associated with an increase in plasma levels of PEA; a significant peak concentration of PEA was found 1 hour after the compound administration, while PEA plasma levels dropped to basal values 4 h after um-PEA administration. These results differ from those previously obtained by Vacondio et al. [36] following the oral administration, to rats, of a formulation of PEA (100 mg/kg suspended in corn oil and subjected to ultrasonication/vortexing). In fact, the authors reported that the administration of PEA induced a 3.5-fold rise in plasma concentration after 60 min followed by a return to baseline within 2 h. The discrepancy between their results and the present findings (higher plasma increase at 60 min and longer duration of effect) could reflect differences in PEA bioavailability related to the different formulations or the different species used (i.e., rats instead of mice). However, a recent study reported an increase in plasma PEA levels lasting at least four hours in carrageenan-injected rats administered with [^13^C]_4_-um-PEA (30 mg/kg) [21], thus supporting the present results. In view of the encouraging results obtained on um-PEA absorption, we next assessed hippocampus and PFC (i.e., two brain regions that are involved in cognitive functions and are particularly affected in AD pathology) levels of PEA after a single oral dose of um-PEA (100 mg/kg). The results indicate increased hippocampus and PFC PEA levels following the oral administration of um-PEA. However, the increase in PEA levels reached the statistical significance only in the hippocampus. These data are in line with previous studies performed by orally administrating um-PEA in rats [21] or subcutaneously injecting a corn oil emulsion of PEA in mice [37], and suggest the ability of um-PEA to cross the blood-brain barrier. Finally, the demonstration that the absorption and brain distribution of um-PEA did not change following eight days of administrations indicates the lack of possible pharmacokinetic adaptation to consecutive um-PEA dosing. Overall, the present pharmacokinetic results suggest that um-PEA has a reasonably good oral bioavailability, and support the suitability of chronic oral um-PEA treatment to target CNS disorders. These findings, together with prior studies demonstrating the in vivo efficacy of PEA in counteracting/reducing AD-like symptoms in animal models of the pathology [15], led us to investigate oral um-PEA effects in 3×Tg-AD mice, by focusing on the hippocampus (i.e., the brain area in which significant increases in PEA levels were observed after the compound oral administration).

Firstly, we found that um-PEA rescued the early learning and memory deficits in five-month-old 3×Tg-AD mice. In particular, following a three months of oral treatment with um-PEA, 3×Tg-AD mice displayed a significant improvement of both short- and long-term memory. These results confirm our previous findings demonstrating clear learning and memory improvements in six-month-old 3×Tg-AD mice chronically treated with um-PEA via a subcutaneous delivery system [22]. According to the present data, other preclinical studies indicated that subcutaneous PEA administration reduced or prevented cognitive impairments in different animal models of AD [38,39]. Interestingly, um-PEA exerts no significant effects on learning or memory in non-Tg mice, suggesting that the compound possibly acts on the molecular mechanisms underlying the reduction of cognition in AD.

It is widely accepted that neuroinflammation and oxidative stress play a critical role in the pathophysiology of AD [5,6,7,8,40]. Increased glial cell activation and oxidative stress are reliably observed in rodent models of AD as well, and have been associated to the neuronal loss observed in AD. Thus, a therapeutic approach targeting not only neurodegeneration, but also neuroinflammation and oxidative stress may prove effective in slowing the progression of the disease. A correlation between PEA anti-inflammatory and neuroprotective activities might be suggested from the results in animal AD models, thus attributing to the compound exclusive properties, especially compared with those of common anti-inflammatory agents [15]. The present study indicates that um-PEA exerts anti(neuro)inflammatory effects following its oral administration. In fact, according with previous findings [22] we measured elevated IL-16 levels in the hippocampus of five-month-old 3×Tg-AD mice, and orally administered um-PEA strongly reduced this increase. Interestingly, IL-16 levels are enhanced in AD patients, confirming that the immune system and neuroinflammatory pathways may play an important role in the development and progression of this neurodegenerative disease [41]. Contrarily to what observed with IL-16, oral um-PEA treatment failed to affect increased plasma or hippocampal TNF-α levels in 5-month-old 3×Tg-AD mice. This finding is relevant considering that several lines of evidence using genetic and/or pharmacological animal model of AD, demonstrate that the pro-inflammatory cytokine TNF-α signaling aggravates both Aβ and tau pathologies in vivo. Accordingly, interventions aimed at contrasting TNF-α induced effects are associated with a reduction in brain pathology and an amelioration of cognitive function in rodent models of AD [42]. The possibility that the failure of um-PEA in reducing TNF-α, observed in the present study, is related to the route of administration (i.e., oral) is unlikely, since similar results were previously observed following the subcutaneous administration of the compound. In fact, chronic subcutaneous treatment with um-PEA almost completely abolished the increase in several inflammatory markers observed in 6-month-old 3×Tg-AD mice, but not iNOS and TNF-α [22]. Overall, the present and the prior findings [19,22,39] suggest that chronic um-PEA restrains neuroinflammation, although some pro-inflammatory markers are not affected by the compound. The relevance of this observation remains to be elucidated in further studies. 

In contrast to the several evidence demonstrating PEA-induced anti(neuro)inflammatory properties in AD animal models [15], there are no data on the possible involvement of antioxidant mechanisms in the beneficial action of PEA against the progression of the pathology. This is possibly due to previous data suggesting that PEA lacks direct antioxidant activity to prevent formation of free radicals and to counteract damage to DNA, lipids and proteins [43]. Other results indicate, however, that the compound enhanced the antioxidant capacity of the hippocampus in an animal model of depression [44] and decreased oxidative stress in a mouse model of diabetic peripheral neuropathy [45]. Moreover, it has been reported that PEA exerts a protective effect against in vitro plasma lipid peroxidation and displays protective effect against oxidative damage [46].

The possibility that PEA could exert antioxidant activities in AD pathology seems to emerge, for the first time, from the present study. We firstly observed elevated basal ROS production in 5-month-old 3×Tg-AD mice, when compared to non-Tg animals. This finding is in contrast with a previous study, demonstrating that basal ROS production is similar in wild-type and 6-month-old 3×Tg-AD mice [47]. Although other causes cannot be excluded, this discrepancy may be explained by the fact that oxidative stress is an early event in AD-like progression in 3×Tg-AD mice. In fact, the levels of serum oxidized proteins were found to be increased in three-month-old 3×Tg-AD mice compared with the non-Tg control mice, whereas no significant differences were observed in 6- and 12-months-old AD mice [48]. Based on the author’s findings, it seems likely that in five month-old 3×Tg-AD mice, as those used in the present study, a small increase in basal ROS production is still measurable. Interestingly, the oral treatment with um-PEA fully counteracted this increase, suggesting that the compound could exert direct or indirect antioxidant effects in this animal model of AD. This view is also supported by the fact that, as part of its transcriptional activity, PPAR-α (i.e., a molecular target of PEA) also induces the expression of genes implicated in mitochondrial and peroxisomal fatty acid β-oxidation, and peroxisomes have a crucial role in the metabolism of ROS and lipids, and their importance in brain physiopathology is well established [49]. Interestingly, it has been reported that PEA, by increasing allopregnolone synthesis through PPAR-α in astrocytes, exerts antioxidant activity in vitro [50]. Thus, it could be speculated that these events take place in the brain of um-PEA-treated 3×Tg-AD mice as a consequence of increased PEA levels. Other mechanisms, however, cannot be excluded.

The evidence for anti(neuro)inflammatory and antioxidant properties of oral um-PEA, prompted us to also evaluate whether the chronic oral um-PEA treatment was associated with neuroprotection. To this aim, the synaptic marker synaptophysin was determined in the hippocampus of 3×Tg-AD and non-Tg mice. Unfortunately, we failed to observe significant differences in synaptophysin levels between 3×Tg-AD and non-Tg mice, although a trend to a decrease was measured in the genetic model of AD. Although these findings could have been expected in light of the lack of massive neuronal loss in the cortex and the hippocampus of 3×Tg-AD mice [23], one prior study reported significant reduced synaptophysin levels in the cerebral cortex of this animal model of AD) [51]. Furthermore, a decrease in the expression of synaptophysin was observed in 3 months-old 3×Tg-AD mice associated with a trend to a compensatory increase relative to that in wild type animals in a topographic and time-dependent manner [52]. The treatment with um-PEA did not affect the marker levels in both genotypes, thus suggesting that under the present experimental conditions oral um-PEA treatment failed to exert neuroprotective effects. However, since no baseline changes were found between transgenic mice and non-transgenic controls, it might be possible that under these experimental conditions um-PEA cannot further affect these processes. Further experiments are necessary to fully investigate on these aspects.

Unbalances in the glutamatergic system have been well documented in AD patients [53,54], while increased glutamate levels and abnormal NMDA receptor activation have been associated to excitotoxicity in AD [55]. In line with this view and according to our prior results [22], in the present study we observed a marked increase in the levels of extracellular glutamate in the hippocampus of 3×Tg-AD mice, probably due to a reduced expression (~+35%) of a glial transporter mainly responsible for glutamate reuptake (GLT-1) observed in 3×Tg-AD mice at the same pathology stage as those used in the present study [22]. In contrast with findings previously obtained from subcutaneously um-PEA treated 3×Tg-AD mice [22], in the present study we demonstrate that the oral treatment with um-PEA significantly reduced this increase, although failing to restore control (i.e., non-Tg mice) levels. Interestingly, a recent study demonstrated that um-PEA treatment fully counteracts the increase in glutamate levels observed in neuropathic mouse dentate gyrus [56], an effect possibly associated with reduced excitotoxicity and cognitive impairment. These data suggest that um-PEA, possibly by reducing excitotoxicity, could contrast neurodegenerative processes in the used transgenic model of AD and rescue cognitive impairments. For the sake of clarity, however, it should be considered that in this study we did not observed significant signs of neurodegeneration from synaptophysin analysis and, as a consequence, the above hypothesis remains to be confirmed in further studies. The discrepancy between the present and our previous results might be due to the fact that in the present study, compared to the prior ones [22], a higher dose of um-PEA has been used (100 and 10 mg/kg/day, respectively).

## 5. Conclusions

Overall, this study reinforces the concept that um-PEA exerts a marked therapeutic effect in 3×Tg-AD mice, ameliorating both cognitive deficits and a range of neuropathological features. In particular, also based on previous findings [15], it seems likely that um-PEA treatment by reducing neuroinflammation and oxidative stress could contrast brain damage, thus rescuing cognitive deficit in 3×Tg-AD mice. Furthermore, the reduction of the elevation in extracellular glutamate, could also contribute to the effects of um-PEA on cognitive function in this animal model of AD. Interestingly, um-PEA treatment also counteracted the glutamate increase likely associated with the recovery of LTP induction in neuropathic mouse [55], thus ameliorating the cognitive decline-associated loss of neural plasticity in this animal model.

It is worth mentioning, however, that some features of this study present some limitations and may deserve further investigation: relative high dose (100 mg/kg/day) of PEA has been used. In fact, preclinical in vivo studies have demonstrated that the most pharmacologically efficacious effective dose of PEA is 10 mg/kg [57], and we previously demonstrated that the subcutaneous chronic treatment with 10 mg/kg um-PEA is effective in reducing AD-like symptoms in 3×Tg-AD mice [22]. Concerning this aspect, the relatively high dose of PEA has been chosen in the present proof-of-concept study in order to obtain the first clear evidence of efficacy after the compound oral administration. In view of the promising results, we are planning, for translational purposes, to test the compound at lower doses, also in order to render more feasible the use um-PEA to treat AD. It is worth noting that, on the basis of the 100 mg/kg dose used in the present study, a 70-kg human would require several grams of um-PEA. However, based on the usually higher metabolic capability of mice than humans, it seems likely that a lower dose (e.g., 600–1200 mg/day) would be sufficient. To partly support this view, a case report study suggested beneficial effects of um-PEA (700 mg/day), in combination with the antioxidant luteolin, in a patient affected by mild cognitive impairment [58]. Furthermore, a study involving 30 Parkinson’s disease (i.e., another neurodegenerative disease) patients receiving levodopa demonstrated that um-PEA (600 mg for 1 year) slowed down disease progression and disability [59]. As anti-inflammatory um-PEA is clinically used at 600 mg twice a day, while relevant PEA-induced side effects were not seen in humans at oral doses up to 1800 mg/day [15], higher doses not being tested. Finally, it becomes also urgent to determine PEA pharmacokinetics at the end of the chronic treatment, also to provide new insights on the apparent efficacy of PEA, despite its quite short half-life; very preliminary data (*n* = 2/group) suggest that in the hippocampal tissue from um-PEA-treated 3×Tg-AD mice, mean basal PEA levels were higher than those measured in standard diet transgenic animals (201 ± 34 pmol/g and 128 ± 35 pmol/g, respectively; Beggiato et al., unpublished). The presence of possible bias from endogenous PEA and the pitfall relative to the recently reported PEA contamination in glass pipettes and the polyurethane foam used for their packaging [60] could have affected pharmacokinetic results. Possible age and gender differences have not been evaluated. The treatment was performed at an early stage of the pathology, when the animals are mainly at a pre-symptomatic stage of AD-like pathology. For translational purposes, it becomes relevant to also evaluate the effects of oral um-PEA treatment in clearly symptomatic 3×Tg-AD mice. In this context, it is relevant, however, that a chronic subcutaneous administration of PEA to 12-months-old 3×Tg-AD mice induced beneficial effects against AD-like symptoms [22]. The possible role of the modulation of systemic inflammation in the beneficial effects of um-PEA on cognitive-deficit in 3×Tg-AD mice has not been fully investigated. In fact, several findings strongly suggest that systemic inflammation can result in neuroinflammation, thus shaping a cerebral inflammatory milieu that may seriously impact neuronal function. In the present study we only observed that um-PEA treatment failed to modulate the increased plasma TNF-α levels in 3×Tg-AD mice. However, it cannot be ruled out that the compound could reduce other markers of systemic inflammation, thus modulating the systemic and CNS inflammation crosstalk [61].

Despite the above limitations and by avoiding any simplistic extrapolation of data from the animal model to the human condition, the results of the present study, together with previous data [15] suggest um-PEA as a potential therapeutic agent for AD treatment. The fact that um-PEA is already licensed for use in humans as a nutraceutical, food supplement, or a food for medical purposes [14,15], and the demonstrated high safety and tolerability of the compound [62,63], strongly support its rapid translation in clinical practice.
